# Comparison of the consumption of antidepressants in the immigrant and native populations in a Spanish health region: an observational study

**DOI:** 10.1186/1471-2458-10-255

**Published:** 2010-05-17

**Authors:** Inés Cruz, Catalina Serna, Jordi Real, Montse Rué, Jorge Soler, Leonardo Galván

**Affiliations:** 1 Primary Care Research Institute IDIAP Jordi Gol, Catalan Institute of Health, Lleida, Spain; 2 Ronda Health Center, Catalan Institute of Health, Lleida, Spain; 3 Family Medicine Department, University of Lleida, Lleida, Spain; 4 Regional Primary Care Management Office, Catalan Institute of Health, Lleida, Spain; 5 Rambla de Ferran Health Center, Catalan Institute of Health, Lleida, Spain; 6 Pharmacy Unit, Catalan Health Department, Lleida, Spain

## Abstract

**Background:**

Health professionals and organizations in developed countries adapt slowly to the increase of ethnically diverse populations attending health care centres. Several studies report that attention to immigrant mental health comes up with barriers in access, diagnosis and therapeutics, threatening equity. This study analyzes differences in exposure to antidepressant drugs between the immigrant and the native population of a Spanish health region.

**Methods:**

Cross-sectional study of the dispensation of antidepressant drugs to the population aged 15 years or older attending the public primary health centres of a health region, 232,717 autochthonous and 33,361 immigrants, during 2008. Data were obtained from computerized medical records and pharmaceutical records of medications dispensed in pharmacies. Age, sex, country of origin, visits, date of entry in the regional health system, generic drugs and active ingredients were considered. Statistical analysis expressed the percentage of persons exposed to antidepressants stratified by age, gender, and country of origin and prevalence ratios of antidepressant exposition were calculated.

**Results:**

Antidepressants were dispensed to 11% of native population and 2.6% of immigrants. Depending on age, native women were prescribed antidepressants between 1.9 and 2.7 times more than immigrant women, and native men 2.5 and 3.1 times more than their immigrant counterparts. Among immigrant females, the highest rate was found in the Latin Americans (6.6%) and the lowest in the sub-Saharans (1.4%). Among males, the highest use was also found in the Latin Americans (1.6%) and the lowest in the sub-Saharans (0.7%). The percentage of immigrants prescribed antidepressants increased significantly in relation to the number of years registered with the local health system. Significant differences were found for the new antidepressants, prescribed 8% more in the native population than in immigrants, both in men and in women.

**Conclusions:**

All the immigrants, regardless of the country of origin, had lower antidepressant consumption than the native population of the same age and sex. Latin American women presented the highest levels of consumption, and the sub-Saharan men the lowest. The prescription profiles also differed, since immigrants consumed more generics and fewer recently commercialized active ingredients.

## Background

The health of immigrant populations is an issue that has aroused a great deal of interest in recent years. The huge increase in the numbers of ethnically and culturally different populations attending health centres in our geographical setting obliges health professionals and organizations to adapt to this new situation and to respond to the needs of these new users. In general, this process of adaptation is slow.

The psycho-social process of loss and change that immigrants experience has been likened to a process of mourning [1]. It may develop favourably, or it may deteriorate into a pathological process associated with chronic and multiple stress (Ulysses' Syndrome). This means that the mental health of immigrants is a priority research issue. Immigrants cannot be considered as a homogeneous group as regards the risk of mental illness, and indeed many studies suggest that this risk is not systematically present in all immigrant groups; however, in specific circumstances certain factors may favour the development of mental illness and may increase the likelihood that immigrants will seek help at health centres [2].

Studies of the prevalence of mental illness in immigrants come up against a series of obstacles that make the identification and diagnosis of this pathology particularly difficult. These include the understandable fear of detection felt by illegal immigrants, their lack of information regarding access to the health system, and their inability to communicate freely due to linguistic difficulties. To compound the problem, the registration systems in use are not designed for a situation of large-scale immigration; the health staff may know little of the immigrants' cultures, and the traditional psychometric scales used to diagnose psychiatric illness may have little transcultural validity [3-5]. Finally, the cultural background of each ethnic group is deeply involved in the mental health concept, identification and help seeking attitudes, resulting in a great variability of situations. For all these reasons, the reports of the prevalence of mental disorder in this group may be unreliable.

In Spain, where the percentage of economic immigration is high, several studies have investigated mental health in the immigrant population. Pertíñez et al. found no significant differences in the percentages of mental illness in a sample of 112 immigrants and 112 natives seen at a health centre between 1995 and 1997, although 32% of the native-born patients were receiving psychodrugs, compared with 20% of the immigrants [6]; Pardo et al. concluded that the prevalence of depression in a sample of 606 sub-Saharan immigrants was similar to that in the native population, although only 6% of immigrant patients diagnosed with depression received antidepressant treatment [7]. In contrast, Barro et al. found higher prevalence of anxiety and depression in a sample of 27 illegal immigrants in the same region in 2003 [8]. Analysing the most frequent diagnoses for which legal immigrants were attended in psychiatric units, Ochoa et al. found them to be similar to those recorded in native patients: anxiety, adaptive disorders, and depression [9].

In a study conducted in a Spanish health region in 2005, Rué et al focused on differences in the general consumption of drugs between a broad sample of immigrants and native patients. They found lower expenditure in the immigrant sample, a lower percentage of prescriptions dispensed, fewer packages withdrawn and lower prices [10]. These findings support the hypothesis that active ingredients may be prescribed differently in the two groups because newer drugs are often more expensive and therefore generic medications would be more frequently prescribed to immigrants because of their lower cost.

In the light of these findings, we designed the present study to compare the percentages of antidepressant use in the immigrant and native populations in a Spanish health region over a period of a year. As the immigrant sample was large, we also divided them into subgroups according to their area of origin. Finally, we also analysed whether the profile of antidepressant prescription differed between the native and immigrant populations in terms of active ingredients (AI) and the use of generic drugs.

## Methods

Type of study and setting: Cross-sectional, descriptive study of the dispensation of antidepressant drugs (AD) during the 2008 calendar year in a Spanish health region (HR).

### Study population

Population aged 15 years or older assigned to any of the Catalan Health Institute's 21 centres in the health region of Lleida, who were recorded in the Insured Persons Registry (IPR) during the 2008 calendar year. The IPR identifies each insuree using a personal identification code. At the time of the study, 95% of the population on the census roll in the HR were listed in the IPR, received health care and were entitled to drug prescription. In the Spanish public health system, drugs are prescribed free to those over the age of 65 and at a 60% discount to patients under this age. An official prescription is needed to obtain AD drugs. The Health Region's economy is based on agriculture and livestock farming, with the result that immigration is eminently labour-oriented. The proportion of the foreign population has risen from 2.6% in 2000 to 14.1% in 2007 [11]. To assess the representativeness of our sample, the distribution by countries of origin of the population assigned to the health centres was compared with that of the population recorded on the census in the HR.

### Variables

The demographic variables considered were age, sex and country of origin. The administrative variables analysed were the date of entry in the region's health system, contact with the health centre and the number of visits made during the year of the study. As regards AD consumption, the prescription of generic or brand drugs and the active ingredient were recorded, according to the N06A therapeutic subgroup of the Anatomical Therapeutical Chemical (ATC) Classification System [12].

Classification of the population as immigrant/native: individuals born in Spain were considered as natives, and those from countries with medium or low income according to the World Bank web page [13] were considered as immigrants. If the country of origin was unknown, subjects were considered to be natives if they were listed in the IPR prior to 1 January 2003, when the percentage of immigrants in the HR was below 5%.

Subclassification of the immigrants according to area of origin: Foreign subjects were classified into five culturally different groups: Eastern European, Maghribi, Latin American, Sub-saharan African and Other.

### Exclusions

**Figure 1:**
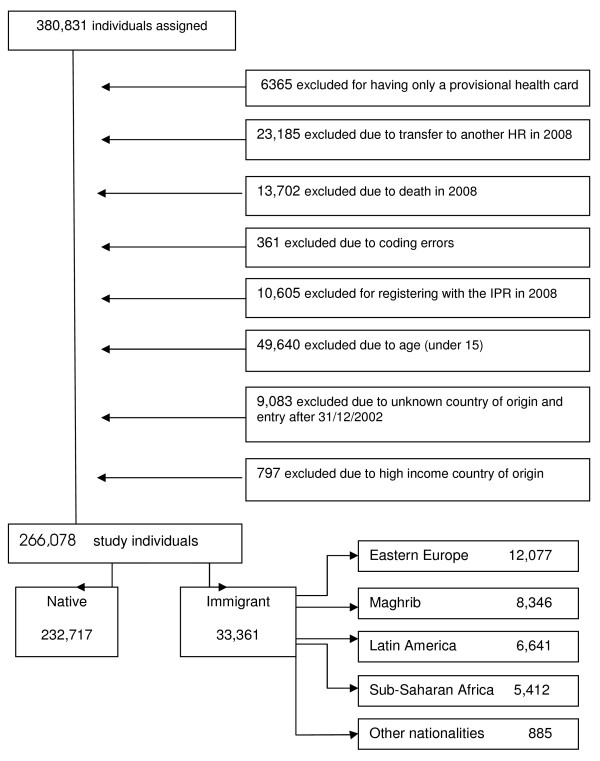
**Selection process of the study population**.

Service users who moved to other areas or who died in 2008 were excluded, as were those whose identification codes were provisional or incomplete, and foreigners from high income countries. The selection process is shown in figure 1.

### Sources of data

Demographic, administrative and clinical data of the study population were obtained from the clinical histories stored electronically in the Primary Care Information System. The population of the HR was obtained from the December 2008 municipal census. The information on patients' drug use was obtained from the prescriptions dispensed at the pharmacy offices, as official prescription is obligatory for medication of this kind. This information was provided by the HR Pharmacy Unit of the Catalan Health Service and was compared with the administrative and clinical data using a common identification code. Permission to use this data was obtained from authorized personnel in the Catalan Health Service and the Directory of Primary Care. All data were treated anonymously to ensure confidentiality. Being an observational retrospective study, no ethical approval was considered necessary.

### Analysis

A descriptive statistical analysis was performed expressing the number and the percentage of users prescribed AD, stratified by levels of the qualitative variables (age group, sex, population and area of origin). The 95% confidence interval (95%) of the rates of prescription and prevalence ratios was calculated using the normal approximation. To evaluate the association between AD prescription and population group we estimated the prevalence ratio of AD use in the native and immigrant populations together with the 95% CI. To evaluate the relationship between AD prescription and the time registered with the health system, the linear trend test of the X^2 ^was used.

We analysed the active ingredient and the use of generic or brand drugs for the subgroup of patients prescribed AD, calculating the percentages stratified by population and sex. The percentages were compared with the X^2 ^test.

## Results

### 1. Description of the study population

#### 1.1. Origin, age and sex

The study population comprised 266,078 individuals. Immigrants from low or medium income countries - Eastern Europe, the Maghrib, Latin America and sub-Saharan Africa - accounted for 12.5%.

The immigrant population was younger (median age 34 years, SD = 10 years) than the native population (median age 49 years, SD = 20 years). The distributions according to area of origin, age group and sex are shown in table 1. The percentage of women varied according to origin, from 58% among the Latin Americans to 20% in the sub-Saharans. Immigrants aged over 65 years accounted for only 1.1% of the total.

**Table 1:** Distribution of the population according to area of origin, age group and sex.

		Latin America		Eastern Europe		Maghrib		Sub-Saharan Africa		Other		Native	
		**n**	**%**	**n**	**%**	**n**	**%**	**n**	**%**	**n**	**%**	**n**	**%**

**WOMEN**	15-24 years	646	16.8	1341	21.4	655	23.8	191	17.6	75	21.4	12496	10.6
	25-34 years	1422	36.9	2509	40.1	1072	39.0	602	55.5	117	33.3	17825	15.1
	35-44 years	1058	27.5	1526	24.4	637	23.2	222	20.5	114	32.5	19816	16.8
	45-54 years	475	12.3	625	10.0	246	9.0	53	4.9	30	8.5	18630	15.8
	55-64 years	144	3.7	193	3.1	84	3.	10	0.9	8	2.3	15885	13.4
	> = 65 years	107	2.8	58	0.9	53	1.9	6	0.5	7	2.0	33453	28.3

													

**MEN**	15-24 years	585	21.0	1004	17.2	678	12.1	459	10.6	85	15.9	13377	11.7
	25-34 years	918	32.9	2218	38.1	2253	40.2	2145	49.6	198	37.1	19170	16.7
	35-44 years	801	28.7	1651	28.3	1842	32.9	1355	31.3	188	35.2	21224	18.5
	45-54 years	347	12.4	764	13.1	636	11.4	318	7.3	53	9.9	19716	17.2
	55-64 years	95	3.4	163	2.8	128	2.3	47	1.1	6	1.1	15472	13.5
	> = 65 years	43	1.5	25	0.4	62	1.1	4	0.1	4	0.7	25653	22.4
	
													

**Total Men**		2789	42	5825	48.2	5599	67.1	4328	80	534	57	114612	49.2

**Total Women**		3852	58	6252	51.8	2747	42.9	1084	20	351	43	118105	50.8

													

**TOTAL by area**		6641	20	12077	36.2	8346	25	5412	16.2	885	2.6	232717	

#### 1.2. Contact with the health system

In all, 90% of the Latin Americans, 84% of the sub-Saharans, 81% of the Maghribis and 70% of the Eastern Europeans on the census roll in the HR were assigned to health centres. The comparison of the distribution of the main cultural groups in both registries was as follows (census versus IPR, in percentages): native, 81.4 vs. 84.9; Latin America, 3.1 vs. 2.9; Eastern Europe, 7.1 vs.5.2; Maghreb, 4.5 vs. 3.8; Sub-Saharan Africa, 2.7 vs. 2.4.

In 2008, 69.7% of the immigrant population studied were attended at primary care centres, compared with 81.6% of the native population. The median annual number of visits was 6 in the immigrant group and 9 in the natives.

Focusing on our subsamples, 74.3% of the Maghribis were attended at primary care centres, 73% of the Latin Americans, 69.6% of the sub-Saharans and 63.2% of the Eastern Europeans. The median annual number of visits was 6 in the first three groups and 5 in the Eastern Europeans.

According to the IPR data, the Maghribi group were the longest established; 20% of its members had spent more than seven years in the HR. The most recent arrivals were the Eastern Europeans, 45.7% of whom had registered in the last two years.

### 2. AD prescription

Ten per cent of the study population (26,513 subjects) withdrew at least one AD from the pharmacy in 2008: 11% of the native population and 2.6% of the immigrant population.

AD use was higher in the native population than in the immigrants in both sexes and in all age groups. Depending on age, native women were prescribed AD between 1.9 and 2.7 times more frequently than immigrant women, and native men 2.5 and 3.1 times more than their immigrant counterparts (table 2).

**Table 2:** Percentage of exposure and risk ratio of AD use in native subjects *versus *immigrants, by sex and age group

		Immigrants	Native	Prevalence ratio
		**%**	**%**	**95%CI**

**WOMEN**	15-24 years	1.86	3.55	1.91 (1.45 2.53)
	25-34 years	3.98	6.52	1.64 (1.43 1.88)
	35-44 years	5.65	10.61	1.88 (1.63 2.16)
	45-54 years	6.30	15.89	2.52 (2.06 3.09)
	55-64 years	7.29	21.14	2.90 (2.07 4.05)
	> = 65 years	9.09	24.23	2.67 (1.77 4.01)

				

**MEN**	15-24 years	0.68	1.72	2.54 (1.60 4.05)
	25-34 years	1.03	3.73	3.60 (2.87 4.54)
	35-44 years	1.54	5.24	3.40 (2.75 4.21)
	45-54 years	1.56	6.44	4.13 (2.93 5.82)
	55-64 years	2.05	8.18	3.99 (2.09 7.64)
	> = 65 years	3.62	11.40	3.15 (1.33 7.44)

AD consumption differed in our immigrant subsamples. Among females, the highest rate was found in the Latin Americans (6.6%) and the lowest in the sub-Saharans (1.4%). Similarly, among the males, the highest AD use was also found in the Latin Americans (1.6%) and the lowest in the sub-Saharans (0.7%) (Table 3).

**Table 3:** Percentage of patients consuming AD according to origin, sex and age group.

		Latin America		Eastern Europe		Maghrib		Sub-Saharan Africa		Native	
		**n**	**%**	**n**	**%**	**n**	**%**	**n**	**%**	**n**	**%**

**WOMEN**	15-24 years	646	3.6	1341	1.5	655	1.7	191	0	12496	3.5
	25-34 years	1422	5.3	2509	4.6	1072	2.8	602	0.8	17825	6.5
	35-44 years	1058	7.7	1526	4.6	637	6.3	222	3.1	19816	10.6
	45-54 years	475	10.1	625	4.3	246	4.5	53	3.8	18630	15.9
	55-64 years	144	11.8	193	4.1	84	8.3	10	0	15885	21.1
	> = 65 years	107	10.3	58	6.9	53	1.9	6	16.7	33453	24.2
	
	**Total Women**	3852	**6.6**	6252	**3.9**	2747	**3.6**	1084	**1.4**	118105	**15.3**

**MEN**	15-24 years	585	1.0	1004	0.7	678	0.7	459	0	13377	1.7
	25-34 years	918	1.3	2218	1.2	2253	1.0	2145	0.8	19170	3.7
	35-44 years	801	2.5	1651	1.7	1842	1.7	1355	0.6	21224	5.2
	45-54 years	347	2.0	764	0.8	636	2.7	318	0.9	19716	6.4
	55-64 years	95	1.0	163	1.8	128	3.1	47	2.1	15472	8.2
	> = 65 years	43	0	25	8	62	3.2	4	25	25653	11.4
	
	**Total Men**	2789	**1.6**	5825	**1.2**	5599	**1.5**	4328	**0.7**	114612	**6.6**

	**TOTAL area**	6641	**4.5**	12077	**2.6**	8346	**2.2**	5412	**0.8**	25651	**11.0**

The percentage of immigrants prescribed AD also increased significantly (p < 0.001) in relation to the number of years registered with the local health system. This was the case in all age groups: when time on IPR was 1-2 years, the percentage of AD use increased from 0. 96 in the youngest to 3.33 in the 55 to 64 years old; in the other end, when the time on IPR was 9 years or more, the percentages for the same age groups were 2.16 and 8.97 respectively.

### 3.Type of drug prescribed

#### 3.1. Generics

Overall, 32.2% of users prescribed AD received generics: 33.4% of women and 29.2% of men (p < 0.001), and 44.2% of immigrants compared with 31.8% of natives. The RR of receiving generics among the immigrants was 1.4 (95%CI 1.29 - 1.5).

The use of generics also varied depending on the area of origin: 47.5% in Latin Americans (95%CI 46.3-48.7), 45.1% in Eastern Europeans (95%CI 44.2-46.0), 42.3% in Maghribis (95%CI 41.2-43.4) and 28.9% in sub-Saharan Africans (95%CI 27.7-30.1).

#### 3.2. Active ingredients

Types of drug dispensed are shown in table 4, presented according to group and sex.

**Table 4:** Number and percentage of patients exposed to different treatments, according to origin and sex.

	Immigrants				Native			
	
	Men		Women		Men		Women	
	
	n = 236		n = 626		n = 7517		n = 18134	
	
	n	%	n	%	n	%	n	%
**N06AA ADT/HETEROC.(1)**	49	20.8	129	20.6	1297	17.2	3341	18.4
**N06AB SSRI(2)**	170	72.0	484	77.3	5515	73.4	13629	75.2
**N06AG MAOI(3)**	0	0	0	0	0	0	1	0.0
**N06AX SNRI/NDRI/SP.NORADREN. SEROT.(4)**	35	14.8	91	14.5	1776	23.6	4105	22.6

(1) Tricyclic and heterocyclic antidepressants

(2) Selective serotonin reuptake inhibitors

(3) Monoaminooxidase inhibitors

(4) Serotonin-norepinephrine reuptake inhibitors/noradrenaline and dopamine reuptake inhibitors/specific noradrenergic and serotoninergic antidepressants

The most frequently prescribed subgroup (above 72% in both populations) were selective serotonin reuptake inhibitors (paroxetine, escitalopram, citalopram and fluoxetine), followed by tricyclic antidepressants (amitriptyline and clomipramine, between 17% and 21%, with higher percentages of the former in immigrants). Clinically significant differences were found for serotonin-norepinephrine reuptake inhibitors (duloxetine and venlafaxine), noradrenaline and dopamine reuptake inhibitors (bupropion and reboxetine) and specific noradrenergic and serotoninergic antidepressants (mirtazapine and oxitriptan), which were prescribed 8% more in the native population than in immigrants, both in men and in women.

The rates of prescription of the various AI differed in our subsamples, with the greatest differences for amitriptyline, especially in the Maghribi and sub-Saharan groups (see table 5).

**Table 5:** Main active ingredients dispensed according to area of origin (numbers and percentages).

	NATIVE		IMMIGRANTS		Latin America		Eastern Europe		Maghrib		Sub-Saharan Africa	
	**n**	**%**	**n**	**%**	**n**	**%**	**n**	**%**	**n**	**%**	**n**	**%**

**Paroxetine**	5793	22.6	260	30.2	85	28.2	113	35.6	44	24.2	11	24.4
**Escitalopram**	5280	20.6	144	16.7	47	15.6	63	19.9	28	15.4	5	11.1
**Citalopram**	3913	15.3	146	16.9	60	19.9	53	16.7	29	15.9	4	8.9
**Fluoxetine**	3226	12.6	98	11.4	46	15.3	30	9.5	18	9.9	2	4.4
**Venlafaxine**	2899	11.3	52	6.0	21	7.0	17	5.4	13	7.1	1	2.2
**Sertraline**	2452	9.6	59	6.8	23	7.6	21	6.6	9	4.9	4	8.9
**Duloxetine**	2067	8.1	45	5.2	15	5.0	18	5.7	10	5.5	1	2.2
**Amitriptyline**	1897	7.4	122	14.2	38	12.6	27	8.5	41	22.5	13	28.9
**Clomipramine**	1442	5.6	32	3.7	10	3.3	7	2.2	14	7.7	1	2.2
**Mirtazapine**	1342	5.2	38	4.4	14	4.7	10	3.2	10	5.5	4	8.9

## Discussion

Antidepressant consumption in the immigrant population registered at the health centres in this Health Region was between 40% and 75% lower than in the native population of the same age and sex (prevalence ratio of native vs. immigrants between 1.64 and 4.1).

### Comparison with other countries

This finding corroborates the differences already reported in countries which traditionally have high rates of immigration. A recent systematic review by Uiters et al. underlined the importance of the effect of the host country and its health system on the use of services by the immigrant population. Those authors found more differences in countries with weaker primary care systems such as the US than in areas with stronger primary health care such as Canada and Europe [14].

In the United States, various studies in broad samples coincide in demonstrating that access to AD is especially limited in Afroamericans [15-17]. Williams et al. analysed data from national mental health surveys for Black Africans, White Americans and Black Caribbeans; although the lifetime prevalence of depression was lower in the Black population, the disorder was more persistent and severe, in spite of the fact that they received less AD treatment [17].

In Europe the prevalence of mental illness has been examined in ethnic minorities, and although less research has been conducted of their pharmacological treatment some studies suggest the existence of differences. One study in the UK, for example, found that Asians received fewer AD and anxiolytics than the rest of the population [18].

As we noted above, studies of mental illness in immigrants have been carried out in Spain, but few compare the rates of pharmacological treatment received by immigrants and natives. Most of those conducted to date are based on interviews of small samples, selected using different criteria which do not allow analysis of the differences between subgroups from different cultures.

### Discussing factors related to the results

In the analysis we included a variety of factors that may have a bearing on the lower use of AD in the immigrant group.

The first point to consider is whether the prevalence of depression is the same across the populations. It has been suggested that there are substantial differences across ethnic subgroups. Unfortunately, the data available from different countries in the world are difficult to compare due to the differences in the diagnostic criteria and instruments applied and in the demographic characteristics of the samples studied. In Spain, the most recent information, from the ESEMeD project (2009), reported a prevalence of major depressive episode above 4% in the last year or 10.6% during the lifetime, rates lower than those found in the other western countries that took part in the project [19]. In Latin America, using similar criteria, yearly prevalence of 4.9% [20] and lifetime prevalence of 9.2% [21] has been reported. In Africa, studies with similar methodologies have also obtained variable yearly prevalence: Nigeria 1.1% [22], Ethiopia 4.4% [23], South Africa 4.9% [24]. Studies in Eastern European countries using different instruments have obtained higher prevalence, ranging from 21% in Poland and 33% in Russia [25]. Finally, there are few data from North African countries, but a study of the prevalence of anxiety and depression in immigrants living in the Netherlands found figures of 10% in Moroccans (and 19% in Turks) compared with 6.6% in the native Dutch population [26]. With occasional exceptions, then, the prevalence of depression in immigrants from these areas is not lower than that found in the Spanish population and does not explain the large difference in treatment, although inter-ethnic differences should be taken into account.

Secondly, the lack of objective data on the prevalence of depression in our immigrant sample means that we cannot evaluate the hypothesis of undertreatment because of underdiagnosis. This hypothesis has been suggested by multiple studies: in the US, for example, Borowsky et al. found that primary care physicians were less likely to detect mental health problems among African Americans and Hispanics than among White Americans [27]. Later studies obtained similar rates of prescription of AD treatment for the three groups, but reported a much lower likelihood that Africans and Hispanics would take the medication than Whites [28]. Although the latter result suggests that GPs are now more alert to the problem and are better at diagnosing depression in countries where the subject has been researched for many years, it does not explain why the differences in AD consumption persist.

Treating patients from other cultures remains a significant problem for primary care physicians in most countries. The obstacles they face include the inadequacy of the diagnostic tools and clinical guides at their disposal and the difficulty of distinguishing between the symptoms caused by traumatic experiences and adverse life conditions and those caused by depression. They may also need to overcome gender stereotypes in mental illness to avoid under- or over-estimation of depressive symptoms [29].

The third factor to consider is the low level of contact with health system [30]. The lower the contact, the lower the levels of diagnosis and treatment. Indeed, in our study, the percentage of immigrants visiting the doctor at the health centres was lower than that of the native population (68.7% vs. 81.6%). These data corroborate the findings of previous studies (e.g. Saura et al., [5]), and have been attributed to the lack of information regarding the health system and of the possibilities of access for those who are not legally resident [3]. In our study, however, all the population included were legally entitled to health care, and had been registered with their health centre for at least one year; therefore, the obstacles would not be to do with the subjects' legal status but with other no less important causes such as linguistic difficulties, cultural differences in the perception of the need to seek medical advice, or the search for alternative solutions to health problems.

Another parameter to take into account in relation to depression is the time already spent in the country. The illness usually emerges as time passes and as immigrants begin to feel that their expectations have not been fulfilled [31]. As we had no precise information on this issue we used an indirect indicator - the date of entry to the health system in the region - as a measure of time flows, of the stability of subjects' residence in the region, and of their adaptation to the local environment over time. Most of the immigrants had arrived in the last four years: the Maghribis and Latin Americans were the most stable groups, although the low numbers of individuals with more than five years' residence means that the results for longer periods should be interpreted with caution. In all age groups, AD consumption increased with time registered in the IPR, supporting the hypothetical model of the healthy immigrant whose health deteriorates with the passing of time. Furthermore, the groups with the highest rates of AD use, the Latin Americans and Eastern Europeans, were not the ones who had spent the longest in the region, a finding that suggests the influence of other factors related to the culture of origin.

Finally, we should stress that the decision to withdraw medication from the pharmacy is taken by the patient. A GP or psychiatrist may prescribe a drug, but the patient's intention to take it may be influenced by a range of circumstances. For instance, immigrant populations generally have low economic resources and the obligation of paying 40% of the price of the drug may be a deterrent. The acceptance of the diagnosis and the pharmacological treatment may also influence the decision to take the medication; in a study in the US, Cooper et al. observed a lower likelihood to accept AD medication in African Americans and Hispanics and a higher prevalence of negative beliefs [32].

### Differences across ethnic groups

The group with the highest AD consumption was the Latin American group, especially the women, and the one with the lowest was the sub-Saharan group, although the samples are too small to be able to generalize the results. Possibly these findings reflect the fact that the cultural and linguistic differences vis-à-vis the study setting are far greater in the case of the sub-Saharans than in the case of the Latin Americans.

In the case of the sub-Saharans, the concept of depression has changed radically in recent times. Historically non-existent or extremely rare, the prevalence of depression in these countries is now equal to or higher than those reported in the developed world, even among different ethnic groups. Recent studies of attitudes to mental illness in low-income communities in South Africa report stigmatization of mental patients, ignorance of the causes, and little effort to seek treatment [33]. A broad survey carried out in 2007 in the US found that ethnic minorities were less likely to believe in the effectiveness of medication or in the biological basis of depression, and more likely to believe in the effectiveness of advice and prayer [34]. Clearly, there is a need for further studies of attitudes to mental illness in these groups.

### Qualitative differences in AD prescription

As regards type of drug, immigrants were prescribed higher levels of generics and lower levels of recently commercialized drugs such as venlafaxine or duloxetine. These results can be attributed to an attempt on the part of physicians to reduce the cost of the medication in patients assumed to have low or zero purchasing power and thus to increase the likelihood of compliance with treatment. We intend to explore this idea further using a cost analysis per patient in a future study.

### Strengths and limitations

One of the strong points of the present study is its large population-based sample and its comparison of the results of immigrants and the native population, with data from 232,717 Spanish nationals, 12077 Eastern Europeans, 8346 Maghribis, 6641 Latin Americans, 5412 sub-Saharans and 885 individuals of other nationalities. As the proportions according to area of origin and sex of the sample matched those published by the Catalan Statistics Institute for the same year, 2008 [11], we believe that our data are representative of the populations of origin, even though the study was performed in a specific geographical area.

The main limitations of the study are due to the selection of the sample. First, immigrants who were not legally registered in Spain were not included, since they do not have an identity card or health or pharmaceutical coverage. For this reason, the results can only be extrapolated to immigrants who are legally registered. Second, to establish whether the immigrant population assigned to health centres was representative of the immigrants in the health region as a whole we compared their distribution with the data from the municipal census. We found that the Latin Americans, Maghribis and sub-Saharans registered at the health centres accounted for between 80% and 90% of those registered on the census, but in the case of the Eastern Europeans the figure fell to 70%. We cannot rule out the possibility of a selection bias, especially in the latter group, as we do not know whether the population that has not contacted the health system differs in terms of mental health from the population studied. Third, we also decided to exclude patients who registered for the first time, moved, or died during the calendar year studied in order to make sure that the observation period was the same for all subjects. Fourth, as regards the country of origin, our decision to classify as native all subjects who did not report their country of origin and who had been living in the study area at the end of 2002, when the proportion of immigrants was under 5%, may have introduced a bias. Equally, in grouping the patients into four areas of origin to increase the power of the analysis we used our own subjective criteria of ethnic/cultural similarity, which are obviously debatable.

We stress that AD prescription does not necessary imply the diagnosis of a depressive disorder, since these drugs may also be prescribed for other health problems [35]; however, this limitation would be expected to affect all the population equally, regardless of their country of origin. Nor does the act of obtaining medication from the pharmacy guarantee compliance with treatment. Establishing the prevalence of depressive disorder in the two groups and assessing compliance with treatment are beyond the scope of this analysis, but there is no doubt that this information would be useful for evaluating the conclusions, and so attempts should be made to determine these variables in future studies.

We are also aware of the possibility of obtaining AD from other sources, but this appears difficult because, in Spain, AD can only be dispensed with a medical prescription and thus would be registered in the database. As far as we know, there are no other organizations in the HR that can provide this service.

Finally, the analysis of AD consumption was performed using an electronic database of prescriptions. Databases of this kind have demonstrated their usefulness for epidemiological analysis of medication consumption patterns in various clinical entities [36-42] carried out from a population perspective.

## Conclusions

All the immigrants, regardless of the country of origin, had lower AD consumption than the native population of the same age and sex.

For all the immigrant population, AD consumption tended to increase with time spent in the health region, reflecting either a deterioration of their health or improved access to the health system.

Latin American women presented the highest levels of AD consumption, and the sub-Saharan men the lowest.

The prescription profiles also differed, since immigrants consumed more generics and fewer recently commercialized active ingredients. This suggests a strategy of cost reduction in order to improve accessibility to medication.

The rather limited bibliography available on the prevalence of depression in immigrants does not explain the clearly lower rates of treatment found in this population. Further studies in specific social and health settings are required to identify and understand the causes of the differences found here between our ethnic subsamples. These studies should focus on specific groups, especially the sub-Saharans, and determine whether immigrants and natives with the same levels of need enjoy the same access to medication and care.

## Competing interests

The authors declare that they have no competing interests.

## Authors' contributions

MCS participated in the study design and helped to draft the manuscript. IC participated in the design, prepared and drafted the manuscript. JR performed the statistical analysis, and helped to interpret the results. MR and JS participated in the design, interpreted the results and reviewed the manuscript. LG provided, validated and helped to interpret pharmacological data.

All authors read and approved the final manuscript.

## Pre-publication history

The pre-publication history for this paper can be accessed here:

http://www.biomedcentral.com/1471-2458/10/255/prepub
